# How Shall We Cure Croup?

**Published:** 1891-04

**Authors:** E. F. Starr

**Affiliations:** Nacoochee, Ga.


### Original Communications.

### HOW SHALL WE CURE CROUP?

By E. F. STARR, M. D., NACOOCHEE, GA.

By this term I mean membranous croup, in contradistinction to the spasmodic form; and in what I shall say on this subject I shall not claim entire originality, but endeavor to bring forward and enforce upon the minds of practitioners some facts that if well observed and carried out will prove a source of utility and comfort to such as may have to treat this formidable disease. If I can present these facts and the method of treatment so as to make them as clear to the minds of those who read as they are to my own, and can enlist general belief in the statement, I shall have accomplished my object and shall have done a good work.

I will not discuss the question of the identity of membranous croup and diphtheria, or whether or not all cases of croup are diphtheritic in character. I do not believe they are, but be this as it may, I shall now use the same treatment in the main for both. During most of the past this disease has been an *opprobrium medicorum*, and that no one treatment is acknowledged and followed as effective and reliable is manifest in the fact that so many different and varying formulas are being successively and from time to time proposed. I am now glad to believe that this state of things need not be perpetuated. In former years I myself regarded an established case of croup as about equivalent to a death warrant, but now I would go about the treatment of a case, not too long delayed, with nearly as much confidence as I would a case of remitting fever with plenty of quinine in my possession.

The pathology and symptoms are too well known to require notice here, but in reference to the treatment let it first be stated what should not be done, for now I eschew almost entirely the practice I formerly most trusted, that is, the administration of emetics, especially that form of them composed of tartar emetic. Do not give them. Persistent emesis is distressing and prostrating to the child, and, except in very rare instances, is ineffectual and unsuccessful. Neither, as a general rule, or scarcely at all, should purgatives be administered in the beginning of the treatment with object of catharsis, for this would interfere with the proper administration and the desired action and effect of the main remedy, the remedy most to be relied on and persisted in.

The indication for the treatment, in my view of the case, is to so affect the blood and the diseased locality as, first, to arrest the continuance of the deposit in the larynx and trachea, and, second, to soften and dissolve or loosen that which has already been exuded, so that it may be expelled by an effort of coughing. Can this be done? My experience teaches me that it can. In past time some of the fathers were known to proclaim that calomel was the sheet-anchor, and I have no doubt they sometime succeeded with it, but not often. How did they administer it? Usually in large doses, hence in purgative doses, and herein was the failure. It was too speedily expelled from the system. They did not, it seems, fully apprehend the philosophy of its effect, that is, of its curative effect. They desired its purgative effect, and it possessed in their eyes a sort of hidden magic. They gave it in purgative doses, but we may suppose in some cases it would remain long enough in the child's stomach to be absorbed and produce the necessary constitutional and salutary effect, and hence their occasional success, enough to make them believe it a useful remedy; but by want of proper manipulation, and by reason of other influences brought to bear against calomel fifty or sixty years ago by a set of arrant quacks and impostors (the Thompsonians), it fell into some little disrepute and failed to be graded into proper line and to be established as *the* remedy for membranous croup. In the hands of the profession it did not grow into the full stature of its inherent capacity, and it is safe to say it has not even done so yet. Let us hope this may not remain true indefinitely. How then shall we proceed to secure its curative effect, since calomel is the remedy ?

In the first place, let it be remembered it is not to be given in purgative doses, for this would prevent its curative effect. It must be given in a way to secure its permeating and modifying effect upon the circulating fluids and the systemic condition; and to this end it should be given in small doses and frequently repeated. A child from one to three years old, after having a dose of two grains (or even three grains if there has been delay), should be given one grain every hour, promptly, persistently and without failure. If any of this one grain is wasted, let enough be added to make up for waste. If these doses incline to purge, add a little paregoric or a drop or two of laudanum to prevent. If a dose is thrown up or rejected, replace with another dose immediately. As auxiliary treatment I usually administer also a febrifuge like this :

℞. Sweet nitre,
Antim. wine,
Syrup ipecac,
Paregoric, aa q. s.

M.—From half to a teaspoonful two to four hours apart.

If there is much febrile excitement, I generally use two or three drops veratrum viride three or four hours apart to restrain the circulation, and in addition to these I use and advise a small blistering plaster over the larynx or upper windpipe. These latter measures are resorted to as precautionary, but the chief reliance is placed in the calomel.

During the first hours of this treatment the symptoms may seem to march steadily on towards suffocation, but if properly administered and persisted in, the physician or friends will usually, in the course of twelve or fifteen hours, have the pleasure of observing a marked change for the better in the progress of the symptoms, the sound of the breathing will indicate a growing looseness in the obstruction, and after this, by an effort of the child—a smart struggle, it may be—the accumulation will be forced up into the mouth and may be wiped out, or perhaps may be swallowed, but in either case greatly to the relief of the patient. It is gratifying, aye, it is simply beautiful, to witness the effect of the treatment, the manner in which the obstruction is broken up, and the change from the condition of impending suffocation to that of comparative freedom of respiration. When this occurs the calomel should be discontinued and some action of the bowels procured. There is but little danger of salivation, but it would be preferable to suffocation. I have not known it to occur.

